# Configuration of circum-Antarctic circulation at the last green- to icehouse climate transition

**DOI:** 10.1073/pnas.2520064123

**Published:** 2026-04-06

**Authors:** Hanna S. Knahl, Johann P. Klages, Lars Ackermann, Katharina Hochmuth, Lu Niu, Nicholas R. Golledge, Gerrit Lohmann

**Affiliations:** ^a^Alfred Wegener Institute, Helmholtz Centre for Polar and Marine Research, Bremerhaven 27570, Germany; ^b^Australian Centre of Excellence in Antarctic Science, Institute for Marine and Antarctic Studies, University of Tasmania, Hobart, Tasmania 7001, Australia; ^c^Antarctic Research Centre, Victoria University of Wellington, Wellington 6140, New Zealand; ^d^Department of Environmental Physics, University of Bremen, Bremen 28359, Germany; ^e^MARUM - Center for Marine Environmental Sciences, Bremen 28359, Germany

**Keywords:** paleoclimate, ocean circulation, oceanic gateways, early Antarctic Circumpolar Current

## Abstract

During the Eocene–Oligocene Transition (∼34.4 to 33.7 Ma), major climatic and tectonic changes initiated Earth’s current icehouse climate. Plate motion intensified mountain building, reduced atmospheric CO_2_, and triggered global cooling. Crucially, the opening of the Tasman Gateway and Drake Passage initiated the formation of the Antarctic Circumpolar Current (ACC), today Earth’s strongest ocean current. However, the ACC’s initial structure during its onset remains poorly understood, limiting our understanding in controlling global circulation patterns and heat distribution at the time. Here, we present data-validated, high-resolution coupled climate–ice sheet simulations of the Early Oligocene Glacial Maximum (∼33.7 to 33.2 Ma), showing that early ocean–atmosphere circulation around Antarctica was closely tied to Southern Ocean gateway geometry and the presence of the early Antarctic Ice Sheet. Unlike today, however, these gateways were not aligned with the westerly wind belt, hence limiting ACC development and promoting strong Antarctic amplification. We conclude that orogenesis, CO_2_ drawdown, and Southern Hemisphere gateway opening alone were insufficient to establish a strong ACC ∼34 Ma. Only the later alignment of westerly winds with open oceanic gateways enabled the enhancement of interhemispheric overturning circulation, promoted carbon uptake, and the long-term stabilization of Earth’s icehouse climate.

## Significance

Around 34 Ma, during the Eocene–Oligocene Transition, Earth transitioned from a warm “greenhouse” to our current “icehouse” climate. While the opening of ocean gateways around Antarctica is often credited with enabling the Antarctic Circumpolar Current (ACC) and driving this climate shift, our simulations reveal a more complex story. We show that gateway opening alone was insufficient—the ACC could only develop its climate-cooling effects once westerly winds later aligned with these gateways. Our study uses a data-validated model combining atmosphere, ocean, and ice sheet interactions to reveal high-resolution proto-ACC circulation and its impact on the heat transport toward Antarctica.

The Eocene–Oligocene Transition (EOT; ∼34.4 to 33.7 Ma) represents Earth’s last drastic climatic reorganization from late Paleogene greenhouse to Cenozoic icehouse conditions, coincident with a strong decline in atmospheric CO_2_ concentrations from ∼1,000 ppmv in the late Eocene to ~600 ppmv in the Early Oligocene Glacial Maximum (EOGM; ~33.7 to 33.2 Ma) just after the transition (1). Mainly, intensified orogenesis—such as the build-up of the Himalayan, Andes, Trans-Antarctic Mountains, and European Alps—as well as changes in Southern Ocean circulation patterns led to increased CO_2_ uptake (2–5), global cooling, and glacial inception in Antarctica (6), eventually paving the way for the modern icehouse climate (1).

Increase in the seafloor spreading rate across the Southern Ocean shifting Australia and South America away from Antarctica promoted the opening of Southern oceanic gateways (7), i.e., the Tasman Gateway and Drake Passage, initiating circum-Antarctic water exchange between the Atlantic, Pacific, and Indian Ocean. Today, the Antarctic Circumpolar Current (ACC) is Earth’s strongest ocean current, an integral part of the global ocean circulation, thermally isolating the Antarctic continent from warmer lower latitudes (8, 9) with important stabilizing effects on the Antarctic Ice Sheet (AIS). Currently, however, the warming climate leads to a strengthening and southward migration of the ACC with relatively warm deep waters more effectively reaching the AIS margin (10–12), resulting in increased ice mass loss (e.g., refs. 13 and 14).

Also during the Early Oligocene Glacial Maximum (EOGM) the Southern Ocean Circulation, Antarctic glaciation and CO_2_ were strongly interconnected by complex feedbacks. Under pre-ACC Southern Ocean conditions heat was transported toward the Antarctic margin via large subpolar gyres (e.g., ref. 15). Therefore, the onset of the ACC was a fundamental change for Antarctic environmental conditions and global heat distribution. However, the early circum-Antarctic circulation, as well as its interaction with the AIS during the EOGM (6), is still largely unknown. Some studies propose ACC onset as a trigger for Antarctic glaciation (16, 17), while more recent studies rather conclude a preceding permanent Antarctic ice cover (e.g., refs. 6, 18, and 19). The onset of the ACC is further associated with enhanced carbon pump activity through upwelling resulting in atmospheric CO_2_ decline, global cooling, and stabilization of the icehouse climate (5, 20).

The development of the early ACC seems to be strongly controlled by Southern Ocean gateway depths and their alignment with the westerly wind belt (e.g., refs. 15, 20, and 21). For both gateways, the timing of the opening as well as the timing of the deepening is difficult to constrain since different geological processes acted simultaneously in these regions (e.g., refs. 7 and 22). The Tasman Gateway may have already been open and considerably deep during the EOT (e.g., refs. 15 and 23) and its latitudinal position is relatively well constrained (24), while the timing of Drake Passage opening and deepening still remains strongly debated (e.g., refs. 25 and 26). Therefore, the timing of the onset of the ACC and the shape of its preceding circulation is an ongoing research effort. Seismic and sedimentological studies are giving temporally well constrained but only very local impressions of current presence and strength in the Southern Ocean (4, 20, 27–33). Modeling studies are often restricted to low resolution (17–19) or they do not include interactive earth system components such as the atmosphere, ice sheets, or land surface (15, 21), or are of low complexity only (34).

Here, we disclose comprehensive insight into the EOGM configuration of circum-Antarctic circulation with a data-validated and high-resolution Earth System Model (ESM) that includes a dynamic AIS component (35).

## Westerly Winds Do Not Align with Gateways

We performed a coupled ice sheet-climate simulation with EOGM continental configuration and 840 ppm atmospheric CO_2_ concentration and two reference climate simulations with modern continental configuration, PI ice sheets and CO_2_ levels of 280 ppm (preindustrial, PI) and 840 ppm (PI3). The CO_2_ concentration of 840 ppm is representative for the EOT and serves as upper bound for the EOGM according to recent reconstructions (1). The ocean component of the EOGM simulation has a horizontal resolution of up to 20 km in the Southern Ocean.

**Fig. 1.**
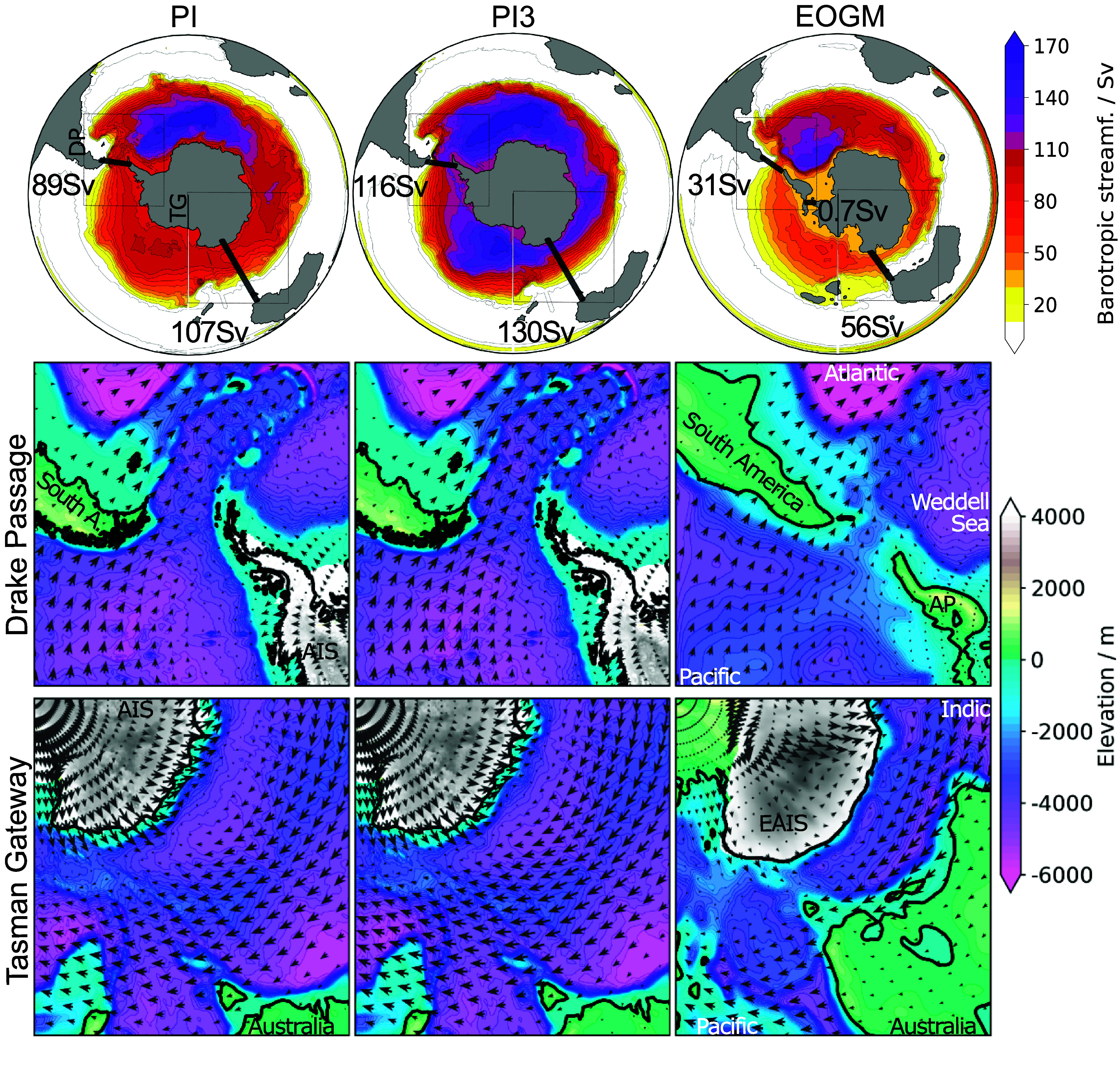
Southern Ocean circulation strength and wind forcing through the Southern Gateways for the EOGM and the reference simulations. (*Top*) The barotropic streamfunction and integrated volume transport through the Southern gateways show a weak proto-ACC for the EOGM (with tripled PI CO_2_) compared to the preindustrial references with 280 ppm CO_2_ (PI) and tripled CO_2_ (PI3). The wind (arrows) within Drake Passage (mid) and Tasman Gateway (*Bottom*) reveal weak or even absent wind forcing in the deepest parts of the EOGM gateways (*Right*), while strong westerlies align with the deeper and wider gateways in the PI simulations (bathymetry is shown in blue-pink shades). Antarctica is fully glaciated in both PI simulations with ice approaching both Southern Gateways (ice sheets are shown in gray shades). The EOGM simulation has a large East Antarctic Ice Sheet (EAIS) but no ice sheet covering West Antarctica. The Antarctic Peninsula (AP) is separated from the continent in the EOGM by a shallow Trans-Antarctic Seaway with a throughflow of 0.7 Sv from the Weddell Sea to the Pacific sector.

When comparing both PI simulations, we identify the westerly wind belt migrating south, toward the Southern Gateways, and strengthening with tripling the atmospheric CO_2_ concentration (Fig. 1 and *SI Appendix*, Fig. S2). Hence, these westerlies force the ACC through the Drake Passage more effectively. Also, the latitudinal density gradient increases across the flow in the higher CO_2_ simulation (*SI Appendix*, Fig. S1) due to changes in buoyancy fluxes, mainly an increased latitudinal gradient in fresh water fluxes (*SI Appendix*, Fig. S2). Together, the stronger density-driven flow and the intensified wind-induced Ekman transport create a stronger ACC (18) and increase gateway throughflow to about 130% relative to PI conditions with 280 ppm (Fig. 1). This effect is observed in proxy and observational records (12) and our model has been shown to perform well in capturing these observations (36).

In contrast, the EOGM simulation develops generally weaker westerly winds resulting in weaker wind forcing through Drake Passage and even absent westerlies in the deepest part of the yet narrow Tasman Gateway. Under identical CO_2_ levels, the resulting volume transport through the gateways is only about 35% of the PI strength (Fig. 1).

**Fig. 2.**
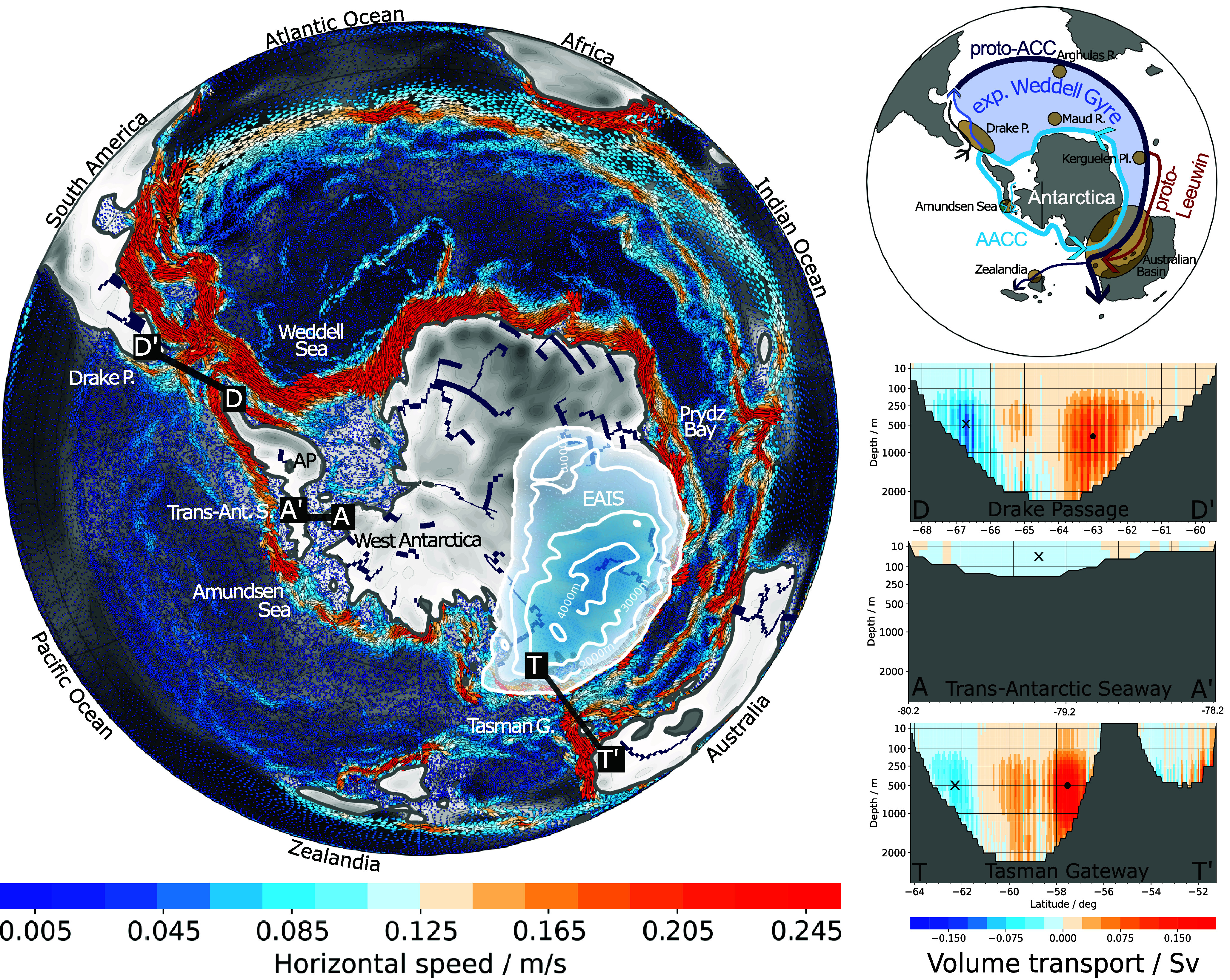
Southern Ocean circulation during the EOGM. (*Left*) Horizontal ocean surface velocity (colors: speed; arrows: direction) averaged over the upper 100 m, overlaid on EOGM bathymetry. A proto-Antarctic Circumpolar Current (proto-ACC) is visible in the Atlantic and Indian sectors, while no continuous ACC exists in the Pacific sector. After passing through the Tasman Gateway near the East Antarctic Ice Sheet (EAIS; ice thickness indicated by white-to-blue shading), the eastward current deflects northward. River systems (dark blue) sourced in the mountains of Dronning Maud Land (topography in white-to-gray shading) feed fresh water into a strong Antarctic Coastal Current (AACC) that flows from the Tasman Gateway toward the Drake Passage. Together the AACC and the proto-ACC form an expanded Weddell Gyre. (*Top Right*) Overview of the dominating surface currents in the EOGM simulation (arrows) and areas we use for the proxy data-model comparison (orange circles). (*Bottom Right*) Ocean volume transport across key transects: Drake Passage (D, D’), Trans-Antarctic Seaway (A, A’), and Tasman Gateway (T, T’). The vertical structure highlights the proto-ACC (red) and the coastal current (blue). Transport through the Trans-Antarctic Seaway is compara tively weaker.

Though the ice sheet is smaller in the EOGM setup—only covering East Antarctica (Fig. 2, *Left*)—while both reference simulations use the PI AIS, we still expect the same effect of higher CO_2_ strengthening the westerly wind forcing by an increased poleward temperature gradient (12). So, under declining CO_2_ concentrations during the Early Oligocene (1), we expect the wind forcing within the gateways and over the open ocean to weaken and, therefore, the proto-ACC to be even weaker than in the shown simulation with 840 ppm.

In the EOGM simulation the westerly winds drive a strong ocean current through the South Atlantic and Indian Oceans reaching the Tasman Gateway but not extending into the Pacific Ocean (Fig. 2, *Left*). As the current approaches the Tasman Gateway, it must shift poleward to higher latitudes compared to its position in the Indian Ocean. Instead of completing to a full circumpolar current after passing the Tasman Gateway, the partial proto-ACC splits up, turns north and dissipates along Australia’s and Zealandia’s East coasts. We explain the northward deflection of the proto-ACC after its entry into the Pacific sector by potential vorticity conservation principles. The potential vorticity, the sum of planetary and relative vorticity divided by the depth, must be conserved along the fluid trajectory. The planetary vorticity of the proto-ACC becomes more negative along its trajectory as it migrates poleward forced by the narrow Australian basin. Further, the ocean depth decreases toward the Tasman Gateway. To balance both changes in potential vorticity, the relative vorticity increases and gives the current a counter-clockwise spin. Consequently, the flow turns equator-ward, once it is no longer constrained to high latitudes by the regional bathymetry. This northward trajectory experiences minimal disruption from wind forcing because westerly winds are absent both within the Tasman Gateway region and in the western Pacific sector beyond it (Fig. 1, *Bottom Right*).

**Fig. 3.**
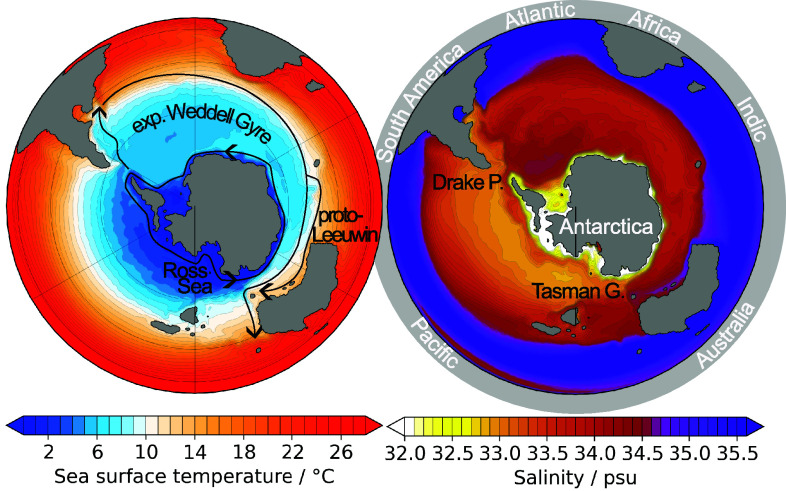
Sea surface temperature with main currents from Fig. 2 (*Left*) and sea surface salinity (*Right*) in the Southern Ocean for the EOGM simulation, highlighting key differences between the Weddell and Ross Seas. The expanded Weddell Gyre exhibits relatively uniform temperature and salinity fields with warm temperatures, exceeding 6 ^°^C, and saline waters. In contrast, the Ross Sea displays colder and fresher waters with greater spatial variability. Cold (below 4 ^°^C) and low-salinity waters are only prevalent along the Antarctic coastal margin. This temperature field has been validated with proxy data in ref. 6.

The whole Pacific sector experiences weak wind forcing (*SI Appendix*, Fig. S2), possibly due to a weaker atmospheric temperature gradient due to the absent WAIS, and a weak latitudinal density gradient (*SI Appendix*, Fig. S1), due to low surface salinity (Fig. 3 and *SI Appendix*, Fig. S2), which both hinder a proto-ACC to establish here (18). In turn, ACC induced upwelling of dense deep waters is absent in the Pacific sector. The resulting strong stratification together with a low surface poleward salt transport, due to the weak Ross Gyre, keep the latitudinal density gradient low potentially hindering ACC establishment.

The EAIS—its position was validated by drill core data presented in ref. 6—influences the proto-ACC by modulating the westerly winds. The ice sheet cools the Antarctic continent and, therefore, increases the temperature gradient that increases the strength of the westerly wind forcing (37). In our EOGM simulation the westerlies are stronger in the Australian basin—close to the EAIS—than in the Drake Passage (Fig. 1, *Right*). But the catabatic winds coming down the EAIS hit the Tasman Gateway in the opposite direction of the westerlies weakening the wind forcing in that critical part of the circumpolar flow. Consequently, the current cannot maintain its initial strength without continued wind stress forcing.

Both Southern Gateways reach a maximum depth of about 2,000 m in our EOGM setup (Fig. 2, *Bottom Right*), which is shallower than the about 4,000 m deep gateways in the PI reference simulations, but they are well below the critical depth for substantial throughflow of 300 m suggested by a sensitivity study for both gateways (15). The applied EOGM gateway widths are about 8^°^ (Tasman Gateway) and 9^°^ (Drake Passage) and as such only slightly narrower than the modern Drake Passage (∼11^°^). The volume transport transects through the EOGM gateways (Fig. 2, *Bottom Right*) show that both, the proto-ACC (red) and its countercurrent AACC (blue), can develop in both gateways. Therefore, we assume the gateway depth and width are not the main restriction to ACC development in the EOGM simulation but rather the driving stresses.

The latitudinal position of the Tasman Gateway used in our model is well constrained for the EOGM (24), therefore, our model results support previous findings indicating that the onset of a complete ACC is only possible once Australia migrates further north to a position where the westerly wind belt and the Tasman Gateway become latitudinally aligned (20, 21, 32).

As suggested by other studies (18, 19), our simulation develops East Antarctic glaciation (6) without a fully developed proto-ACC, hence before the thermal isolation of Antarctica by the ACC. We further showed in a previous study (6) that the ice sheet inception is strongly tied to precipitation induced by warm waters approaching the East Antarctic coast via the proto-Leeuwin current (Fig. 2, *Top Right*). The strong sea surface temperature gradient within Tasman Gateway (Fig. 3, *Left*) is exceptional around Antarctica and has previously been data-validated for this model (6). The bathymetry of the narrow Tasman Gateway plays a key role for bringing this warm current close enough to the continent to allow sufficient precipitation for forming an ice sheet nucleus close to the gateway. The strong uplift of the Transantarctic Mountains in Northern Victoria Land during the EOGM revealed by ref. 38 even beyond the here applied reconstruction (39) make glacial inception in this region even more likely due to cooling of the region.

The glaciation of West Antarctica in the same setup seems to be initiated once atmospheric CO_2_ drops below the critical threshold of 560 ppm (6). Therefore, we assume that the decline in CO_2_ and regional topography are more important to the AIS advance than the thermal isolation by the ACC. Still, the ACC onset and associated upwelling have the potential to strongly promote Southern Ocean carbon uptake by enhancing carbon pump activity, and therefore to sustain global cooling and Antarctic glaciation (5, 20). Since our model does not simulate a full circumpolar proto-ACC for the EOGM, we assume a strong Southern Ocean CO_2_ uptake and its associated climate-cooling effects did not begin until long after the EOGM.

## Circum-Antarctic Circulation Before the ACC Onset

Our simulation reveals detailed coastal currents and an expanded Weddell Gyre dominating the Southern Ocean during the EOGM (Fig. 2). In addition to the recently published data-model comparison for the same simulation used here, which includes air and ocean temperatures, vegetation, and ice sheet cover of Antarctica and its surrounding oceans (6), we here compare the modeled circulation against proxies revealing past ocean current direction and strength. We perform this data-model comparison for key locations throughout the Southern Ocean highlighted in Fig. 2, *Top Right*.

The partial and weak proto-ACC is complemented by a strong westward Antarctic Coastal Current (AACC) which is particularly pronounced between Prydz Bay and the Antarctic Peninsula (AP) (Fig. 2, *Left*). Both currents are connected by a strong northward current that crosses the Drake Passage and together they form an expanded Weddell Gyre (see sketch in Fig. 2). Evidence for a strong and persistent subpolar gyre in the Weddell Sea and weak throughflow through Drake Passage was found in dinocyst assemblages and sea surface temperature reconstructions for the EOT east of the Drake Passage (28, 29).

The expansion of the Weddell Gyre far into the Australian Basin in the model is coherent with sediment drift bodies from both margins of the Australian Basin that confirm increased clockwise circulation toward the late Eocene (32) and align with indications for an enhanced AACC in the Australian basin at ∼34 Ma (30). The expanded Weddell Gyre creates divergence at the surface that leads to upwelling between the opposing currents serving as potential explanation for local high bioproductivity found e.g. in the Australian Basin (4) and along the Agulhas Ridge, Kerguelen Plateau, and Maud Rise (5). Hence, the vertical mixing in the Atlantic and Indic sectors is more pronounced in the EOGM simulation than in the PI reference simulations (*SI Appendix*, Fig. S3). However, as the proto-ACC does not extend far beyond the Tasman Gateway, convection is almost absent in the entire EOGM Pacific sector.

The eastward flow component of the expanded Weddell Gyre that enters the Australian basin is split into two streams—the proto-ACC and a warm coastal current. We associate the coastal component with the warm proto-Leeuwin current (compare Figs. 2 and 3), which has also been interpreted from sediment records (30, 31). This current transports warm surface waters toward the Antarctic continent (Fig. 3, *Left*), where—in concert with a strong uplift of the Transantarctic Mountains in Northern Victoria Land (38)—it nucleated the Antarctic ice sheet (6).

The proto-Leeuwin current and the proto-ACC rejoin when passing the Tasman Gateway. Once they reach the Pacific, the eastward flow weakens and approaches Zealandia (Fig. 2). The current along Zealandia’s east coast in our model is similar to the one that is assumed to have created the drift deposits found along the east coast of New Zealand. Its sedimentation history is associated with proto-ACC onset between 36-34 Ma and a more pronounced ACC only well after the EOGM (27).

The pronounced westward AACC that surrounds the entire Antarctic continent could be enhanced by the strong salinity gradient all around Antarctica’s coast (Fig. 3, *Right*) with fresh Antarctic coastal waters sourced by ice sheet and river runoff (Fig. 2). Small portions of the AACC (0.7 Sv) even flow through a shallow Trans-Antarctic seaway directly toward the Amundsen Sea which therefore faced complex ocean dynamics during the EOGM. A sediment drift body serves as evidence for a southward inflow into the Trans-Antarctic Seaway (33). Our model also simulates southward inflow in the particular region close to the Antarctic Peninsula and the northward Trans-Antarctic current emerges further west. But the southward current was associated with an eastward coastal current. Although we see a westward AACC our model might still be able to capture the drift body.

Motivated by these multiproxy data validations, our simulation of the EOGM Southern Ocean current configuration may serve as a basis for future data reconstructions on and around the Antarctic continent and as a testbed for paleoecosystem evolution under CO_2_ and gateway configurations different to present-day.

## Antarctic Amplification

**Fig. 4.**
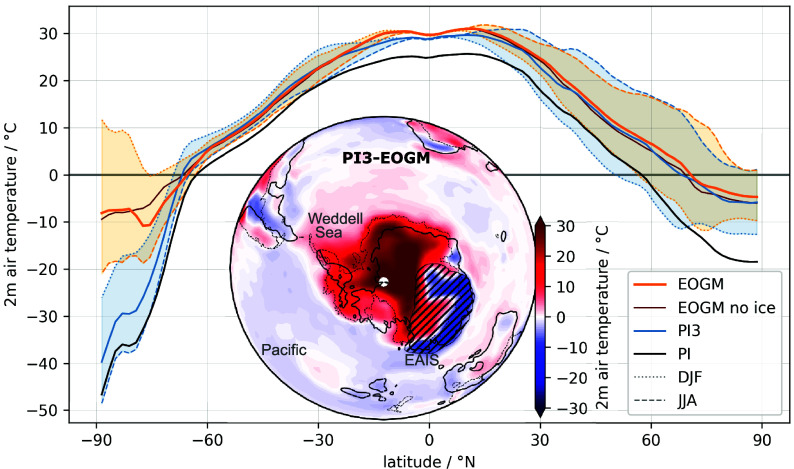
(*Outer*) Annual zonal mean 2 m air temperatures (solid lines) reveal much stronger Antarctic amplification for the EOGM simulations (EOGM no ice refers to the EOGM simulation without AIS) compared to the reference simulation with increased CO_2_ (PI3), while Arctic amplification remains of similar amplitude. The seasonal (dashed/dotted lines) signal shows Antarctic summers (December, January, February–DJF) above freezing point except for the AIS latitude and stronger Antarctic seasonal variability (shaded between DJF and June, July, August–JJA) compared to PI3. (*Inner*) Temperature anomalies of the EOGM simulation relative to PI3 show the main contribution to the Antarctic amplification in the EOGM simulation comes from unglaciated regions in Antarctica. Also above the Weddell Sea air temperatures are increased while they are cooler in the Pacific sector. The Antarctic continent in the EOGM reconstruction (solid line) and with it the EAIS (hatched) is shifted toward Australia relative to the PI position (dashed).

While Arctic amplification during the EOGM simulation reaches similar magnitudes to that under preindustrial conditions with tripled CO_2_, Antarctic amplification is markedly stronger (Fig. 4, *Outer*). We attribute this asymmetry primarily to the ice–albedo feedback and elevation lapse rate effect of the smaller ice sheet. In contrast to the fully glaciated Antarctic continent under PI conditions, the ice-free, vegetated West Antarctic region during the EOGM (Fig. 4, *Inner*); validated by vegetation data presented in ref. 6) reduces surface albedo and increases the absorption of incoming solar radiation. Consequently, also the seasonal variability in the EOGM simulation is enhanced (Fig. 4).

The strongest zonal temperature difference between the EOGM simulations with and without AIS occurs between 70^°^ and 80^°^S where the EAIS develops. The South Pole remains ice free with positive summer temperatures. Moreover, the absence of a fully developed ACC during the EOGM creates a heterogenous oceanic heat transport pattern across the different Southern Ocean sectors. While the Pacific sector is generally cooler than in the PI3 reference, the Weddell Sea and Australian basin allow for greater thermal connectivity between Antarctica and lower latitudes (Figs. 1 and 4). The expanded Weddell Gyre transports warm, saline waters—and potentially enhancing upwelling—along the Antarctic margin (Fig. 3; cf. refs. 4 and 15). Together, these mechanisms establish a more symmetric interhemispheric temperature pattern (Fig. 4; see also ref. 9).

The strength of the westerly wind belt, which is modulated by the equator-to-pole temperature gradient (37), critically governs both the development of the ACC and its thermal isolation of the Antarctic continent. Our results suggest that Antarctic amplification during the EOGM was embedded within a complex feedback system involving atmospheric circulation, ocean gateways, and surface boundary conditions—dynamics that warrant further sensitivity studies beyond the scope of this work.

## Robust Model for Warm Climate Conditions

The final EOT and EOGM represent the most recent periods in Earth’s history to have experienced sustained atmospheric CO_2_ levels beyond 600 ppm—comparable to extreme future emissions scenarios. Yet, direct analogies must be drawn with caution. The Southern Hemisphere circulation during the EOGM was fundamentally shaped by a unique tectonic gateway configuration, which profoundly influenced ocean currents and climate feedbacks. We therefore conclude that, despite similarities in CO_2_ concentrations, the EOGM climate constitutes a nonanalogue state for future warming projections.

Nevertheless, comparisons to modern conditions offer valuable insights into the structural differences between past, present, and possible future climate regimes. We use a comprehensive simulation of circum-Antarctic circulation including interactive components of the ocean, atmosphere, ice sheet, and land surface for the end of Earth’s last dramatic climate shift from green- to icehouse conditions. Our comprehensive validation of the simulation with available data reconstructions verify the robustness of the model for this period of climate reorganization, which directly increases the model’s reliability for predicting future change. We show sensitive interconnections between the position of the westerly wind belt relative to the Southern Gateways, the Southern Ocean circulation and the Antarctic climate conditions. Understanding these interconnections is critical for providing profound climate projections under high CO_2_ emission scenarios.

## Materials and Methods

### Climate Model with Dynamic Antarctic Ice Sheet.

**Fig. 5.**
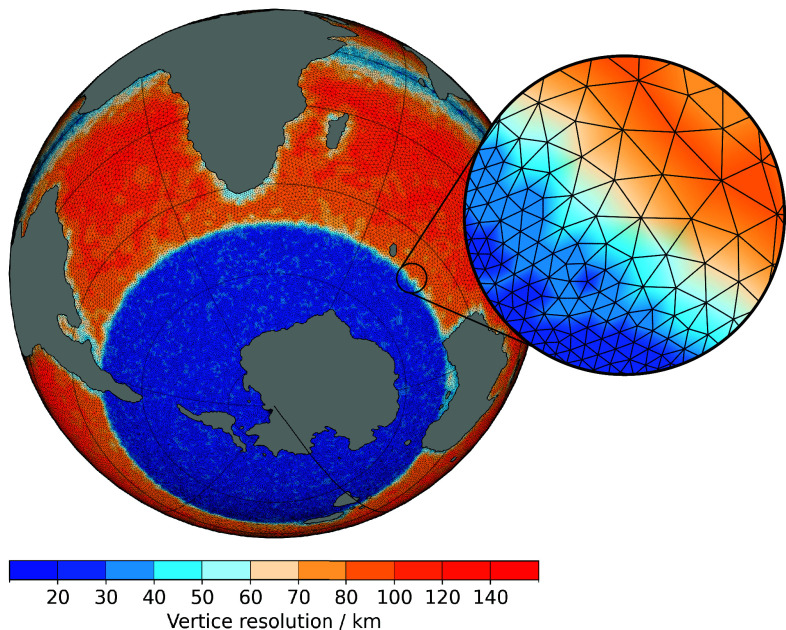
FESOM triangular mesh with variable horizontal resolution for the EOGM with highest resolution in the Southern Ocean.

We use the Alfred Wegener Institute Earth System Model (AWI-ESM, version 2.1) coupled to the Parallel Ice Sheet Model [PISM, version 1.2.1 (40)] to simulate the global climate and AIS during the EOT. The ocean-sea ice component of AWI-ESM [FESOM, version 2.1 (41)] simulates global ocean dynamics on an unstructured prismatic mesh with the highest horizontal resolution in the Southern Ocean (20 km) and variable resolution (20 to 180 km) on other latitudes (Fig. 5). The atmospheric general circulation is modeled by ECHAM6 which couples diabatic processes and large-scale circulations (42). ECHAM6 uses the T63 setup that corresponds to a horizontal resolution of approximately 1.9^°^. The vertical resolution is L47 which resolves the atmosphere up to 0.01 hPa in a hybrid sigma-pressure coordinate system applied on a Lorenz grid (42). Atmosphere and ocean exchange heat and water fluxes via the OASIS3 coupler (43). Land surface and vegetation is modeled by JSBACH, a modular land surface scheme (44) which is directly coupled to the atmosphere model. The AIS is simulated with the Parallel Ice Sheet Model (PISM) and asynchronously coupled to AWI-ESM by regridding the necessary input fields to the corresponding grids (see ref. 35). The ESM-tools manage the technical framework, such as configuration and compiling (45).

### Topography and Bathymetry.

The FESOM mesh and the input files for ECHAM and JSBACH are based on a reconstructed topography and bathymetry for the EOGM (continental configuration shown in Fig. 5) with a resolution of 0.1^°^. It is composed of three reconstructions for different regions. For Antarctica we chose the minimum reconstruction by ref. 39. Most differences to the other reconstructions by ref. 39 are observed in West Antarctica. We chose the reconstruction with the least land above sea level in this area with an open Trans-Antarctic seaway, as this aligns best with recently collected sedimentary data (6, 46) proposes that major parts of West Antarctica already lie below sea level and show potential for open Trans-Antarctic seaways during the EOGM. The corresponding reconstruction of East Antarctica is consistent with the sparse available datasets (39). The Antarctic continent and the updated reconstruction of the Southern Ocean by ref. 7 were integrated into the global paleobathymetric reconstruction by ref. 47 assuring the alignment of plate kinematic reconstruction. We chose the global paleobathymetric reconstruction by ref. 47 as it was particularly interested in updating the gateway reconstruction in the Northern Hemisphere to ensure good gateway reconstructions crucial for this study. Both Southern gateways, particularly interesting for this study, are open in this reconstruction with a width of about 2^°^ and a maximum depth of 2,000 m, shallower than the present-day (Fig. 1). The Drake Passage is shifted Northward and the Tasman Gateway is much narrower than in the present-day due to the proximity of the Australian continent.

### Model Simulations.

The EOT model simulations were carried out in three sequential steps: initialization of the climate system, initialization of the Antarctic Ice Sheet (AIS), and the final coupling of both components. In the first step, a climate spin-up was performed using AWI-ESM in stand-alone mode for 500 model years under preindustrial orbital parameters. This spin-up was based on generalized boundary conditions for temperature, vegetation, and hydrology, assuming an atmospheric CO_2_ concentration of 840 ppm (corresponding to 3× the preindustrial level) and incorporating EOGM-specific paleogeography. Ice sheets were not included in this phase, as the climate spin-up served as the forcing basis for subsequent AIS development.

In step two, the AIS was initialized using the PISM in stand-alone configuration, forced by output from step one. This spin-up extended over 100,000 model years to capture long-term ice sheet dynamics. In the final step, results from both spin-ups were combined and AWI-ESM and PISM were asynchronously coupled and run toward quasi-equilibrium (*SI Appendix*, Fig. S4). To reduce computational cost, the coupling was accelerated by a factor of 100, exploiting the different response times of the climate and ice sheet systems—resulting in simulations covering 1,000 climate years and 100,000 ice sheet years.

As reference climate simulations we ran a preindustrial set-up of AWI-ESM with fixed Antarctic and Greenland Ice sheets using CO_2_ levels of 280 ppm and 840 ppm and the standard model configuration (e.g., ref. 48).

### Data analysis.

All figures are based on 50-y mean data, either annually or seasonally averaged.

## Supplementary Material

Appendix 01 (PDF)

## Data Availability

Model input files data have been deposited in Input files for EOGM coupled simulation with AWI-ESM2 and PISM, Zenodo (DOI: 10.5281/zenodo.16422220). Some study data are available: The raw model output is not deposited into a public data repository due to its size. Full raw model output is available upon request from the corresponding author. Previously published data were used for this work [Model code: (49). Analysis tool: https://github.com/FESOM/tripyview].
