# Supplementary material for: Configuration of circum-Antarctic circulation at the last green- to icehouse climate transition
Source: Proc Natl Acad Sci U S A. 2026 Apr 6;123(15):e2520064123. doi: 10.1073/pnas.2520064123 (PMC13080016; doi:10.1073/pnas.2520064123)
Supplement: Supplementary file 1 — Appendix 01 (PDF) [file pnas.2520064123.sapp.pdf]

Supporting Information for

Configuration of circum-Antarctic circulation at the last green- to icehouse climate transition

H.S. Knahl, J.P. Klages, L. Ackermann, K. Hochmuth, L. Niu, N.R. Golledge, G. Lohmann

Corresponding Author: Hanna S. Knahl.
E-mail: hanna.knahl@awi.de

This PDF file includes:

Figs. S1 to S4

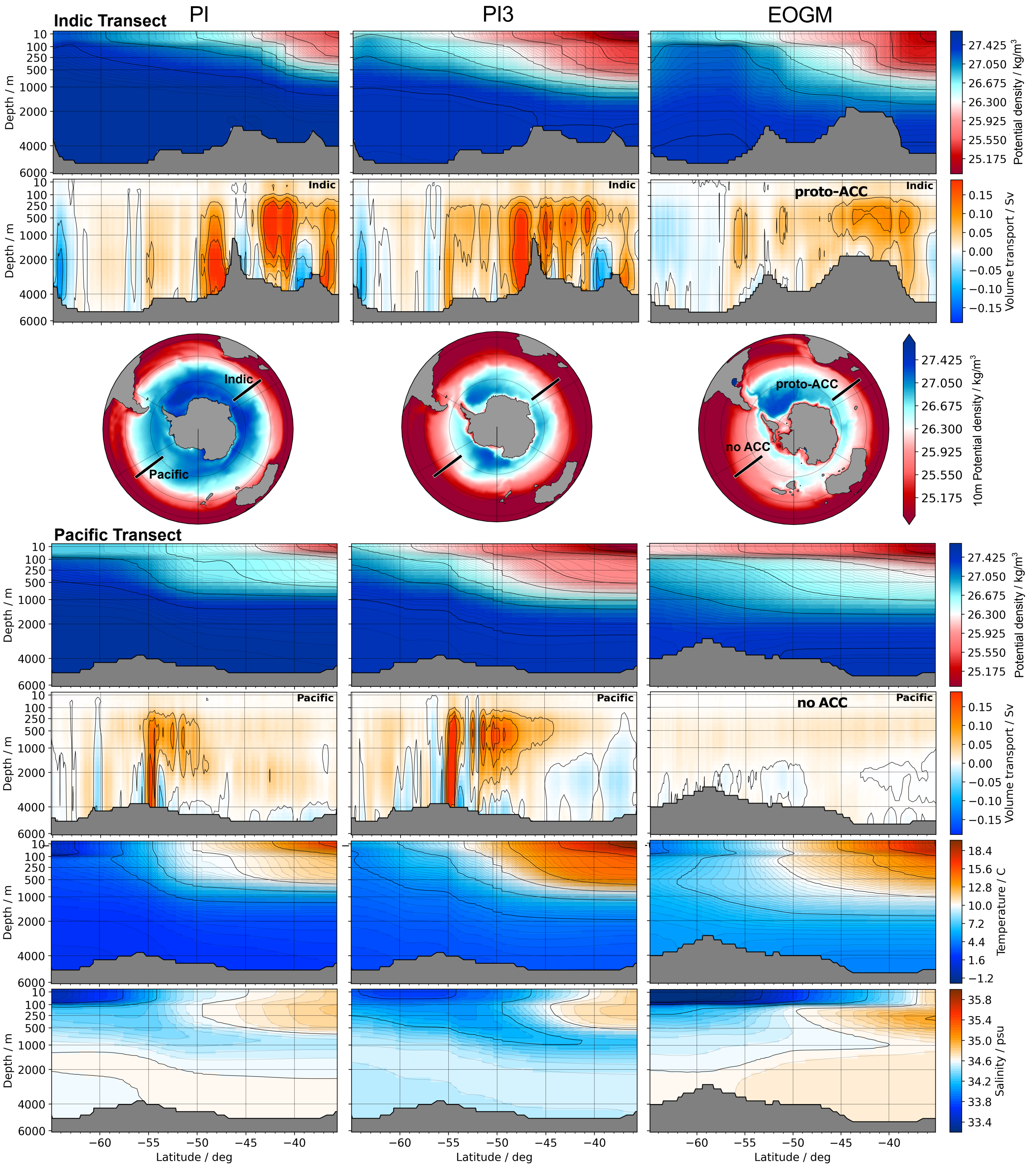

Fig. S1. The potential density in the Southern Ocean (middle) and in latitudinal transects through the Indic (top) and Pacific (bottom) sectors highlight different conditions in the two basins. In the PI reference simulations the latitudinal density gradient increases with higher CO_2 in both basins (Pacific and Indic).
In the EOGM simulations the surface density and the density gradient through the entire water column are much more asymmetric comparing the different ocean basins than in the reference simulations. In the Indic basin, where the proto-ACC is present and the latitudinal density gradient is even enhanced compared to the reference simulations, but the ACC transport is weaker. In the Pacific transect the ocean is stratified very uniformly and the latitudinal density gradient is significantly weaker than in the reference simulations due to low surface salinity.

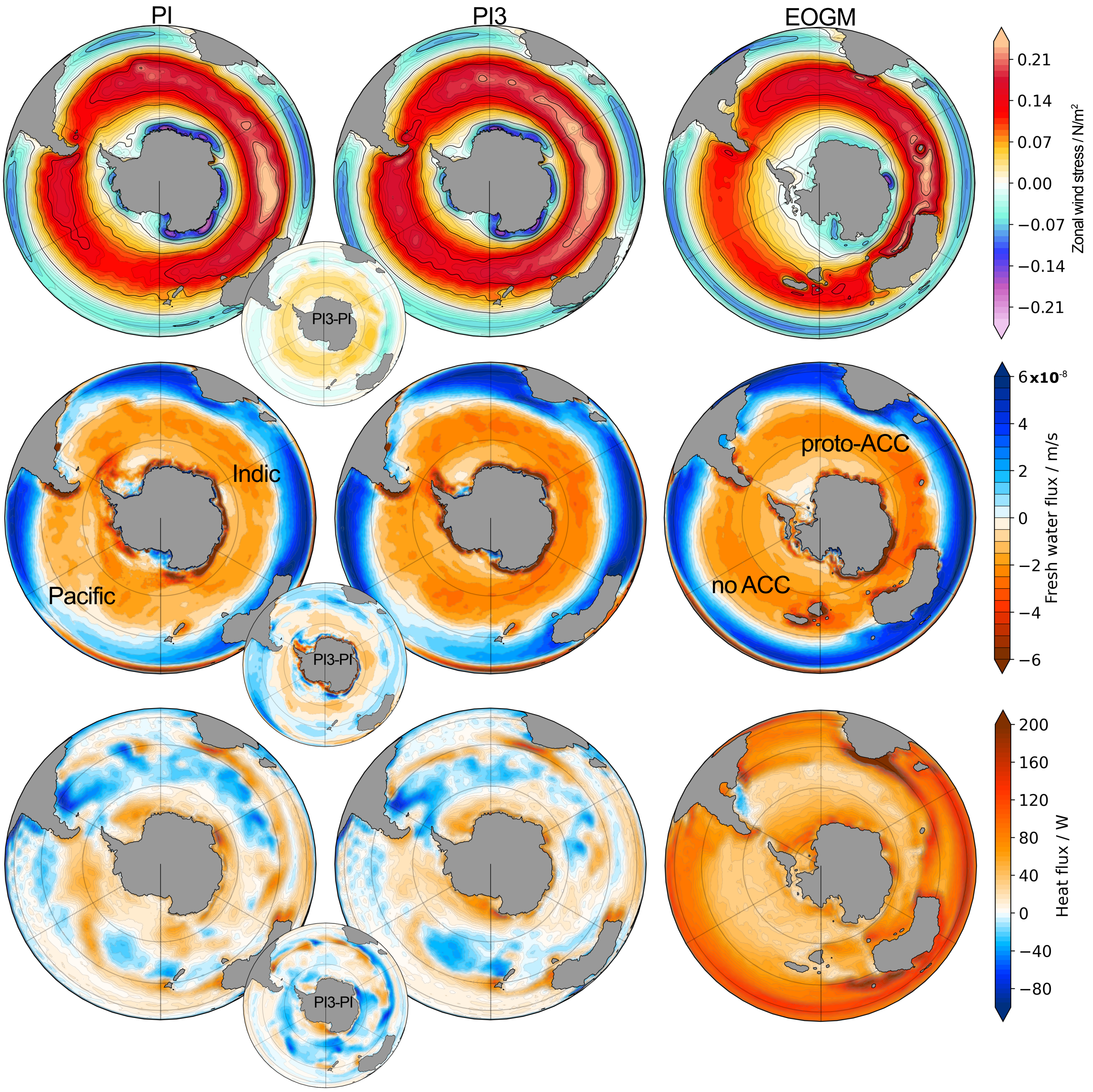

Fig. S2. The zonal wind stress (top), fresh water flux (middle) and heat flux shown for the reference simulations PI and PI3 and the EOGM simulation. In the PI reference simulations the wind stress increases with higher CO_2 in both basins (Pacific and Indic) and shifts poleward (PI3-PI). Also, the latitudinal gradient in fresh water flux increases, enhancing the density gradient (Figure S1). Possibly, also to changes in heat flux contribute to the intensified density gradient, at least for the Pacific basin, though the heat flux pattern is more heterogeneous.

In the EOGM simulation the wind stress in the Indic sector is comparable to the PI but is much weaker in the Pacific sector. No such strong asymmetry can be found in the fresh water and heat fluxes. The fresh water flux pattern and magnitude of the EOGM simulation and the latitudinal gradient of the heat flux are similar to PI3, though the absolute heat flux values are generally higher. Thus, the buoyancy fluxes cannot explain the strong asymmetry in density gradient (Figure S1) between Indic and Pacific sectors.

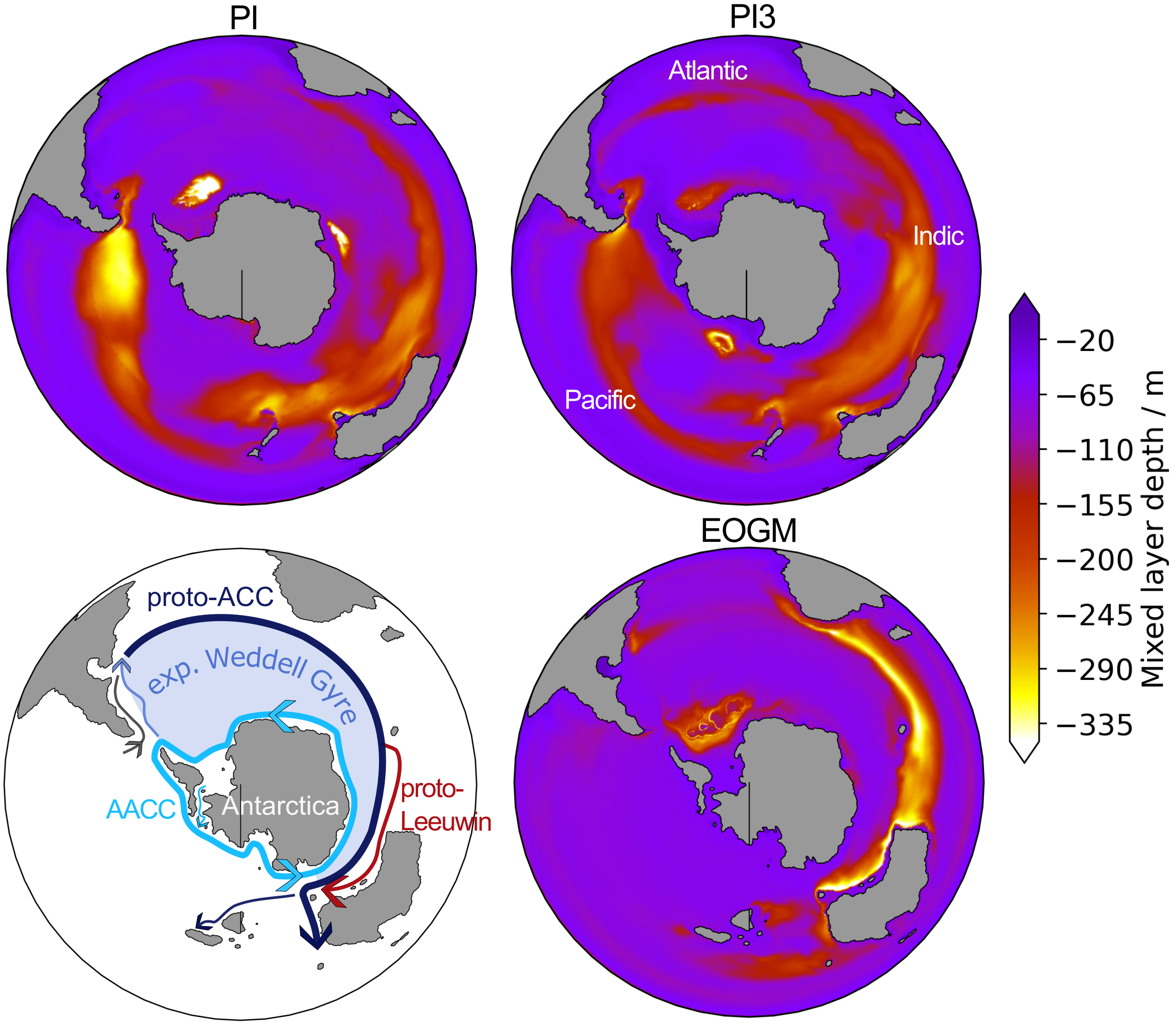

Fig. S3. The mixed layer depths (MLD) in the Southern Ocean show an increase in the Indian sector comparing EOGM (bottom right) to the PI reference simulations (top). Therefore, the proto-ACC in this simulation (sketch) is associated with enhanced convection, whereas MLD is reduced in the Pacific sector where the proto-ACC is absent. Further, strong convection occurs in the Weddell Sea for PI and EOGM whereas it is reduced in PI3.

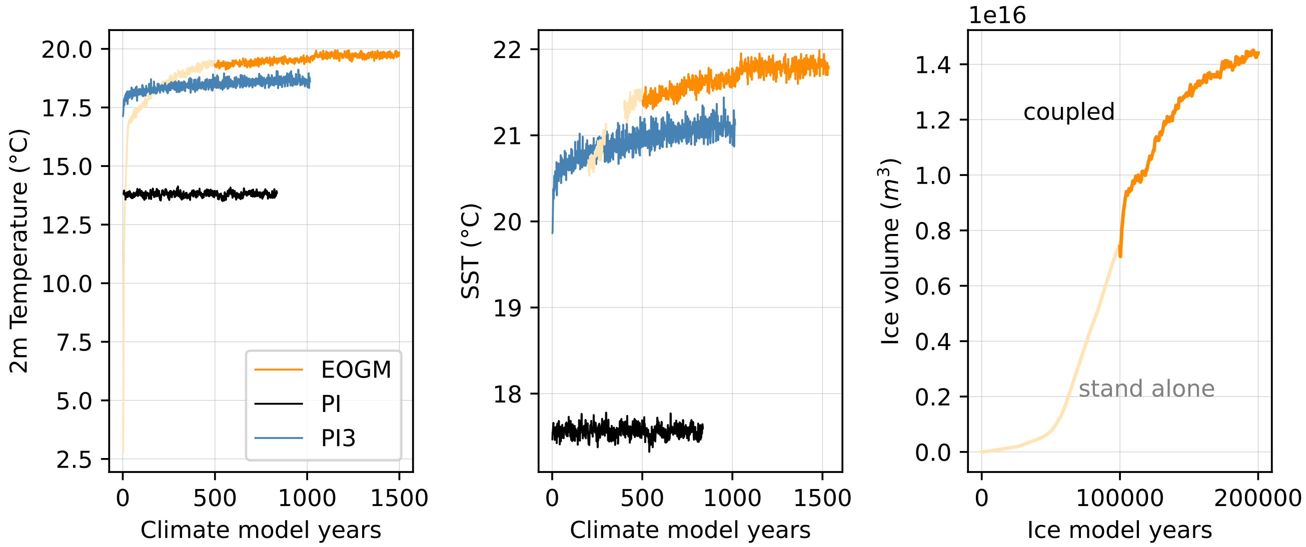

Fig. S4. The temporal evolution of the 2m temperature, sea surface temperature (SST) for all simulations (PI, PI3, EOGM) and the ice volume time series for the EOGM simulation show that all models reached a quasi-equilibrium for the most important variables. The spin ups for the EOGM simulation were run in AWI-ESM stand alone for the climate (light orange in left and middle panel, the SST has two data gaps due to data loss) and PISM stand alone for the ice sheet (light orange). After the spin ups AWI-ESM and PISM were coupled for the EOGM simulation (dark orange).
